# A Methodology for Enhancing SSVEP Features Using Adaptive Filtering Based on the Spatial Distribution of EEG Signals

**DOI:** 10.3390/mi14050976

**Published:** 2023-04-29

**Authors:** Shengyu Wang, Bowen Ji, Dian Shao, Wanru Chen, Kunpeng Gao

**Affiliations:** 1 School of Information Science and Technology, Donghua University, Shanghai 201620, China; 2 Unmanned System Research Institute, Northwestern Polytechnical University, Xi’an 710072, China; bwji@nwpu.edu.cn (B.J.); shaodian@nwpu.edu.cn (D.S.); 3 Innovation Center NPU Chongqing, Northwestern Polytechnical University, Chongqing 401135, China

**Keywords:** steady-state visual evoked potential, adaptive filter algorithm, canonical correlation analysis, EEG

## Abstract

In this paper, we propose a classification algorithm of EEG signal based on canonical correlation analysis (CCA) and integrated with adaptive filtering. It can enhance the detection of steady-state visual evoked potentials (SSVEPs) in a brain–computer interface (BCI) speller. An adaptive filter is employed in front of the CCA algorithm to improve the signal-to-noise ratio (SNR) of SSVEP signals by removing background electroencephalographic (EEG) activities. The ensemble method is developed to integrate recursive least squares (RLS) adaptive filter corresponding to multiple stimulation frequencies. The method is tested by the SSVEP signal recorded from six targets by actual experiment and the EEG in a public SSVEP dataset of 40 targets from Tsinghua University. The accuracy rates of the CCA method and the CCA-based integrated RLS filter algorithm (RLS-CCA method) are compared. Experiment results show that the proposed RLS-CCA-based method significantly improves the classification accuracy compared with the pure CCA method. Especially when the number of EEG leads is low (three occipital electrodes and five non occipital electrodes), its advantage is more significant, and accuracy reaches 91.23%, which is more suitable for wearable environments where high-density EEG is not easy to collect.

## 1. Introduction

A brain–computer interface (BCI) aims to transform the activities of the central nervous system (CNS) into computer language. It enables information transmission between the human brain and computer without using other physiological organization structures [1,2,3]. At present, the brain–computer interfaces are mainly divided into invasive and noninvasive. In the invasive BCI, the microelectrode array is mainly implanted into the human brain [4], while in noninvasive BCI, electroencephalography (EEG) signals on the scalp surface are mainly recorded [5,6]. Visual evoked potentials are electrical activities in the visual cortex caused by external visual stimuli. Regan [7] first published his pioneering work on visual evoked potentials in 1979. Subsequent studies found that when subjects were stimulated with a fixed frequency flicker, the visual cortex would generate EEG signals modulated by the frequency of the stimulation [8]. Studies have shown that when the human gazes at a periodic visual flicker stimulus with a frequency greater than 4 Hz, a periodic response EEG is generated in the brain. Such EEG signals, modulated significantly by the frequency of stimulation, are known as steady-state visual evoked potentials (SSVEPs) [9]. The common SSVEP BCI system judges the screen position of the subject’s gaze through the frequency information carried by the SSVEP. It has stable spectrum and high signal-to-noise ratio [10,11]. SSVEP relies on a steady stream of visual stimuli. However, a flashing stimulus lasts too long and tends to cause visual fatigue, and even epilepsy in severe cases. There are several factors that affect the performance of an SSVEP BCI system, including the size of the SSVEP signal detection area [12,13], different stimulus paradigm selection and optimization parameter methods, stimulus paradigm coding paradigm [14], whether advanced neural network techniques are used in identifying signals [15,16], and whether efficient filtering and classification algorithms are used [17,18,19,20].

The traditional SSVEP signal analysis methods mainly include power spectral density analysis (PSDA) [21,22] and canonical correlation analysis (CCA). PSD is a single-channel analysis method. It classifies the visual stimuli converting the EEG signals from time domain to frequency domain [23,24] and identifies the frequency components. As the current major SSVEP classification method, CCA and its improved versions are widely used. CCA-based methods can calculate the correlation between two groups of multidimensional variables [25]. The CCA method proposed by Lin et al. [26] classifies SSVEP signals by analyzing the correlation between EEG signals and template signals. As a multichannel method, CCA is efficient and robust. Based on CCA, Chen Xiaogang et al. [27] proposed an improved filter bank canonical correlation analysis (FBCCA) method using harmonic components in the signal. A convolutional neural network (CNN) serves as a powerful tool to solve this problem [21]. Zhao Xincan et al. [28] proposed a fast multitarget classification method based on augmented reality steady-state visual evoked potential (AR-SSVEP) using a convolutional neural network.

Steady performance of SSVEPs depends on reliable EEG signals. A high-quality EEG requires more EEG channels and proper electrode placement, which is difficult to achieve in practical application environments [29,30]. The existing classical CCA algorithm has the problem of low accuracy when there is a lack of stimulation time or EEG leads [31,32,33,34]. To solve this problem, we tried to reduce the number of electrode channels required for EEG recording, while ensuring a certain accuracy, so that the SSVEP BCI system is more applicable to a wearable environment.

## 2. Materials and Methods

### 2.1. SSVEP Identify Methods

**Figure 1:**
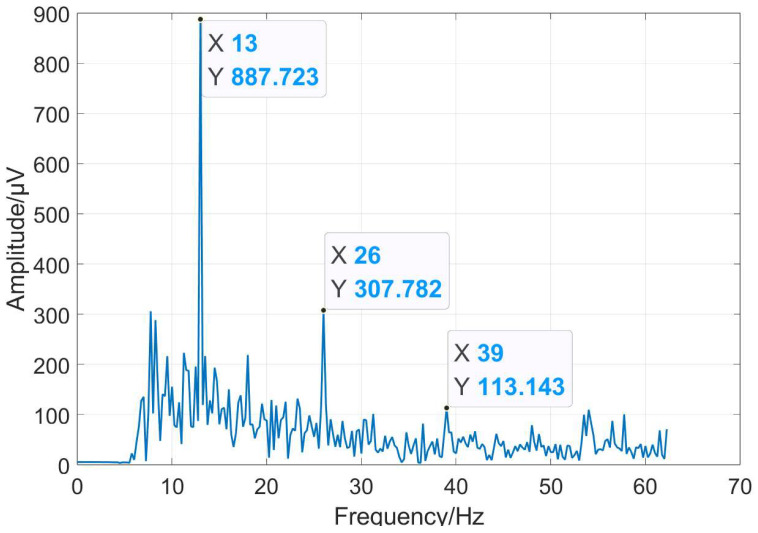
SSVEP response spectrum induced by 13 Hz stimulation frequency.

The EEG signals, SSVEP signals, and nonspecific EEG signals have different characteristics. Figure 1 shows the characteristics of SSVEP signals in the frequency domain, which is the SSVEP response of the subject with a stimulation frequency of 13 Hz. The amplitude of the fundamental component at 13 Hz, the second harmonic component at 26 Hz, and the third harmonic component at 39 Hz are marked. It shows that as the number of harmonics increases, the harmonic energy gradually decreases. SSVEP has the characteristics of time and phase locking in the time domain. Different SSVEP trials will exhibit similar phase characteristics.

**Figure 2:**
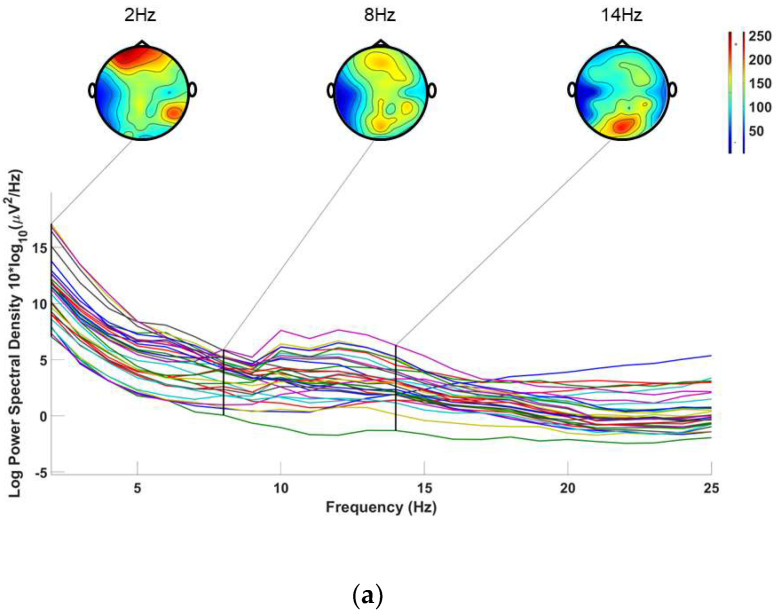

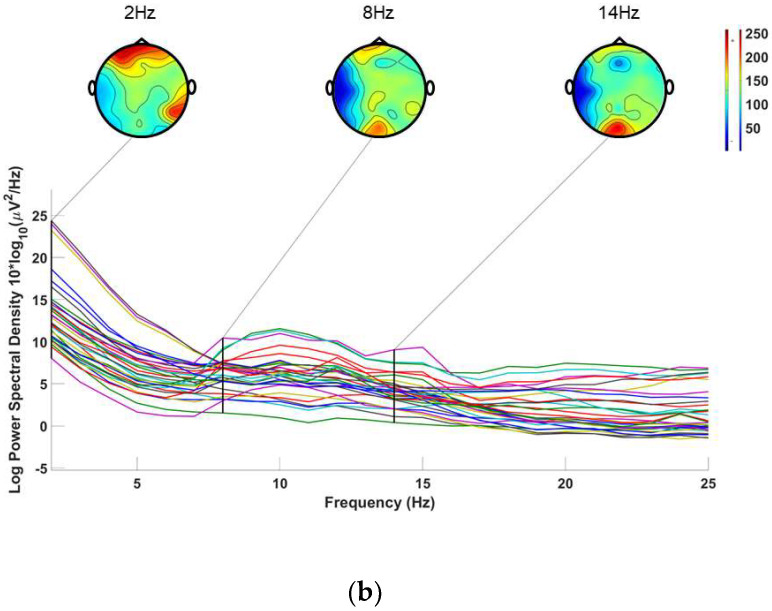
EEG topographic map; (**a**,**b**) are EEG maps of different subjects.

As shown in Figure 2, data were sourced from actual experimental collection. The reference electrode is located in the left earlobe, and the collection device uses an EMOTIV EPOC Flex Pro 32 channel electroencephalogram amplifier (EMOTIV, Epoc flex, San Francisco, CA, USA). All three subjects were subjected to 14 Hz flicker-induced stimulation. In the process of visual induction, 2 Hz also indicates that the higher energy is clearly from other noise disturbances, and the overall energy level of the 8 Hz electroencephalogram is lower; the energy of the occipital lobe was significantly greater at a frequency of 14 Hz in the EEG topographic map at specific time periods.

**Figure 3:**
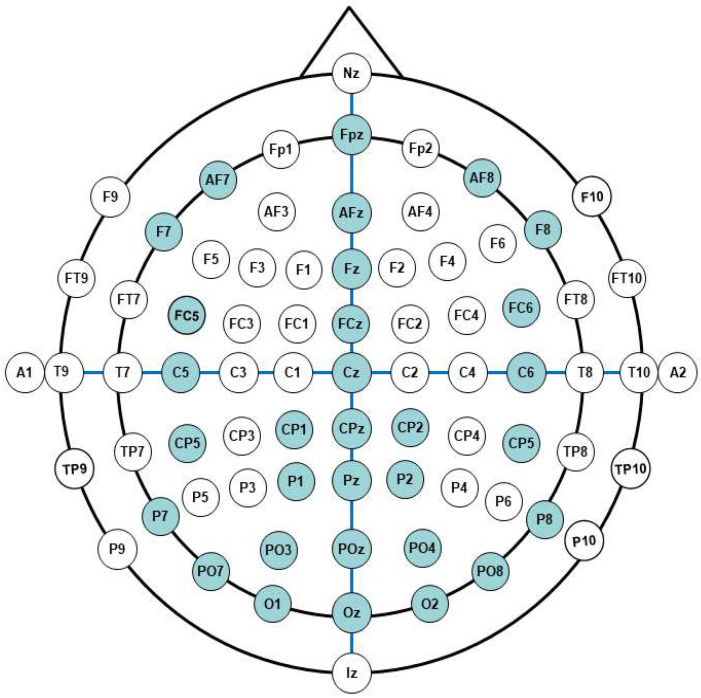
The 10–20 system (EEG).

To further distinguish these features, the common average reference (CAR) [35] algorithm is used to help select useful electrode channels by observing the EEG topographic map. Through this step, fewer electrode channels are selected for EEG recording. Because of the volume conduction effect of scalp EEG, the spontaneous EEG signals and SSVEP specific signals from the nonoccipital lobe will spread to the whole brain region. Similarly, spontaneous and induced signals from other brain regions also transmit to the occipital lobe. Therefore, the noise of mixed signals in the head restraint area will weaken the characteristics of SSVEP. In practical experiments, we add the eye tracker to capture and focus on the target, which allows users to complete the output of the operation commands at any time without prompt, which would be convenient for the user experience. The SSVEP recording system is comfortable, and suitable for the wearable environment. The public dataset used in this paper and the SSVEP data collected in our experiment are recorded from electrodes placed according to the 10–20 system standard, as shown in Figure 3. The 32 channels marked with blue color are selected in actual experiments.

The recursive least squares (RLS) method is applied to extract differences between spontaneous EEG signals and SSVEPs. By extracting this difference, the SSVEP features can be strengthened. RLS [36] is an iterative algorithm that can achieve the best result in each step of the adaptive iterative process. Our purpose is to make the output signal during observation match the expected signal as much as possible in the sense of least squares, which is the noise signal to the occipital lobe of the brain, by selecting the weight coefficient of the adaptive filter. The minimization process needs all available input signal information. In addition, its minimization objective function is deterministic. Adaptive implementation refers to the use of the filter parameters obtained at the previous moment to automatically adjust the parameters at the current moment according to the estimation error, to minimize a certain cost function, and thus achieve optimal filtering. That is, we observe the average power (time average) of the output error signal of the adaptive coefficient of a stationary input signal in a certain time, and take whether the average power is the minimum as the criterion to measure whether the adaptive system is optimal. The specific formulas are as follows:

(1) $$W_{\mathrm{new}\;}\left(n\right)=W_{\mathrm{old}\;}\left(n-1\right)+\Delta W\left(n\right)$$

(2) $$J\left(n\right)=E\left\{|\varepsilon (n){|}^{2}\right\}=E\left\{|d\left(n\right)-W^{H}u(n){|}^{2}\right\}$$

where ΔW(n) refers to the correction item for online data update, d(n) refers to the expected response, u(n) refers to the input signal, and W refers to the least squares estimate of the filter weight vector. The basic steps of RLS algorithm are as follows.

(1) For data initialization:

(3) $$w\left(0\right)=0,R\left(0\right)=\sigma I$$

(2) For data *n* = 1, 2, 3, update the calculation.

Filtering:

(4) $$y\left(n\right)=w^{H}\left(n-1\right)u\left(n\right)$$

Estimation error:

(5) $$e\left(n\right)=d\left(n\right)-y\left(n\right)$$

Update *k* (*n*):

(6) $$k\left(n\right)=\frac{P\left(n-1\right)u\left(n\right)}{\lambda +u^{H}\left(n\right)P\left(n-1\right)u\left(n\right)}$$

Update weight vector:

(7) $$w\left(n\right)=w\left(n-1\right)+k\left(n\right)e^{’}\left(n\right)$$

(8) $$P\left(n\right)=\frac{1}{\lambda }\left[P\left(n-1\right)-k\left(n\right)u^{H}\left(n\right)P\left(n-1\right)\right]$$

**Figure 4:**
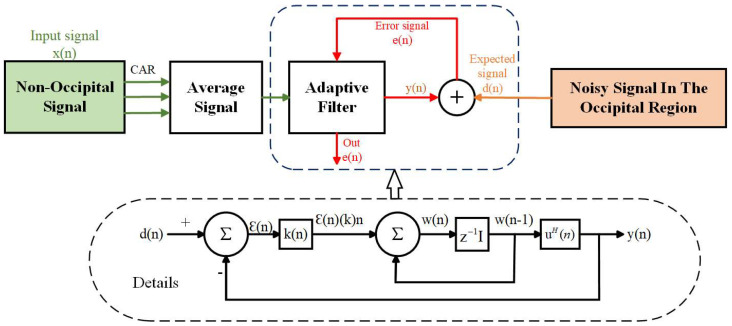
Adaptive filtering process for EEG signal.

We use the RLS algorithm to calculate the actual output of the filter obtained from u(n) at n−1 time using exponential weighting. In practical experiments, d(n) represents the SSVEP signal with noise in the occipital region, u(n) represents the obtained nonoccipital mean signal, $y\left(n\right)$ is the output noise signal, and $e\left(n\right)$ is the pure SSVEP feature signal that is desired. The flow chart is shown in Figure 4.

Then, a CCA step is applied to identify the enhanced SSVEP features. The basic principle of CCA is to analyze the correlation between the multichannel EEG signal set and the template signal set. Combining the previous CAR and RLS filtering algorithms, we obtained two groups of EEG data after processing. Firstly, the representative comprehensive indicators of the two sets of signals are calculated. Then, the correlation coefficient of the two indicators is used to reflect the overall correlation between the two signal sets. Finally, the largest correlation coefficient corresponds to the identified stimulation frequency. The two groups of multivariate variables are defined as X and Y, respectively, where X is the multichannel EEG data processed by RLS adaptive filtering, with higher SSVEP-induced characteristics, and Y is the reference signal in CCA.

Formula (9) is applied to find a pair of vectors for two sets of multidimensional variables, *X* and *Y*, to maximize the correlation between the related variables, where *x =* $X^{T}W_{X}$ and *y =* $Y^{T}W_{Y}$:

(9) $${max}_{W_{X}W_{Y}}\rho =\frac{E\left[x^{T}y\right]}{\sqrt{E\left[x^{T}x\right]E\left[y^{T}y\right]}}=\frac{E\left[W_{X}^{T}XY^{T}W_{Y}\right]}{\sqrt{E\left[W_{X}^{T}XX^{T}W_{X}\right]E\left[W_{Y}^{T}YY^{T}W_{Y}\right]}}$$

**Figure 5:**
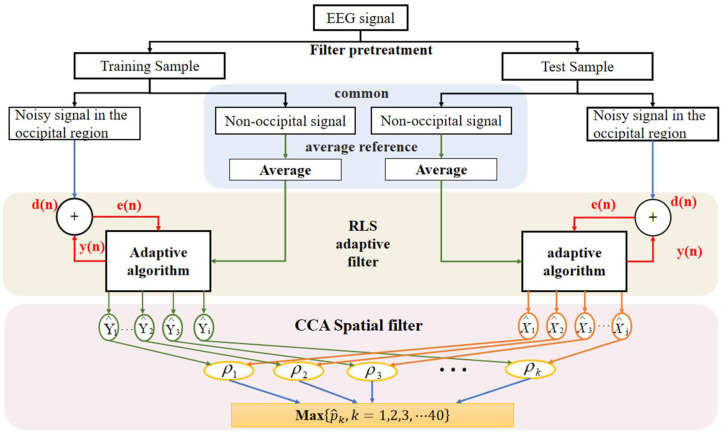
BCI system workflow schematic.

We then calculate the maximum value of the correlation coefficient ρ between *X* and *Y*. After ρ of different f is calculated, the frequency corresponding to the maximum ρ is considered as the response frequency of SSVEP. Figure 5 below shows the schematic of the entire system workflow.

### 2.2. Performance Test Method

#### 2.2.1. Test Based on Public Dataset

To evaluate the effectiveness of the algorithm, this paper used the publicly available dataset from Tsinghua University (THU) for verification [37]. The dataset included 64 channels of EEG data, recorded from 35 subjects (8 experienced and 27 inexperienced) while performing a flicker target selection task. The speller consisted of 40 flickering stimuli with a frequency range of 8 Hz to 15.8 Hz, spaced at 0.2 Hz with a phase difference of 0.5 Hz. They were excited using joint frequency and phase modulation (JFPM) encoding methods. For each subject, the data consisted of six blocks, a total of 40 trials, corresponding to all 40 flashes indicated by visual cues in a random order. The stimulate duration for each test was 5 s.

Training and testing always gives biased results due to random split of data. Here, we used the K-fold cross-validation method [38,39]. The value of K was taken as 6, and in a single classification experiment, the six experiments in the dataset were divided into five experimental groups and one control group. Here, we selected 9 occipital and 55 nonoccipital channels, and used a common average reference algorithm to average the EEG signals of multiple nonoccipital channels, which enhances the spatial difference of EEG and further distinguishes the characteristics of occipital and nonoccipital lobe, visual-induced EEG signals, and nonvisual-induced EEG signals. We continued to reduce the number of electrode channels without reducing the accuracy of signal quality recognition to obtain the pretreated signal. We used an RLS adaptive filtering algorithm to conduct adaptive filtering on the collected preprocessing signals. Finally, through a CCA algorithm, we selected the maximum correlation coefficient as the key inference of target frequency recognition and calculated the accuracy of recognition classification.

#### 2.2.2. Practical Experimental

Pretreatment of the SSVEP experiment was carried out in EEGLAB. Firstly, a 4–30 Hz filter was performed on the collected EEG data, and then the artifact of the processed signal was removed to eliminate the interference of artifacts, such as eye electricity, electromyography, and power frequency interference. We used the independent component analysis (ICA) [40,41] module built in the EEGLAB software to reduce the ocular and myoelectric artifacts in EEG. The experimental group and the control group were divided. In the experiment, we added the detection of events by eye tracker. Through the adaptive filter, the system can detect asynchronously in time and improve the accuracy of feature comparison.

## 3. Experiments Setup

### 3.1. Experimental Paradigm and Procedure

Nine healthy subjects (three females, six males, aged 25 ± 3 years) with normal or corrected vision volunteered to participate in the experiment. Participants read and signed an informed consent form before the experiment. A laptop (ThinkPad E15), a Tobii Eye Tracker 5, and an EMOTIV EPOC Flex Pro 32 channel electroencephalogram amplifier were applied in the experiment. The sampling rate of the EEG signal was 1024 Hz, and all epochs were simply downsampled to 128 Hz. The electrode cap was placed according to the international standard 10–20 system. The reference electrode was located in the left earlobe. The software for data processing was Matlab2021A version (MathWorks, Natick, MA, USA).

**Figure 6:**
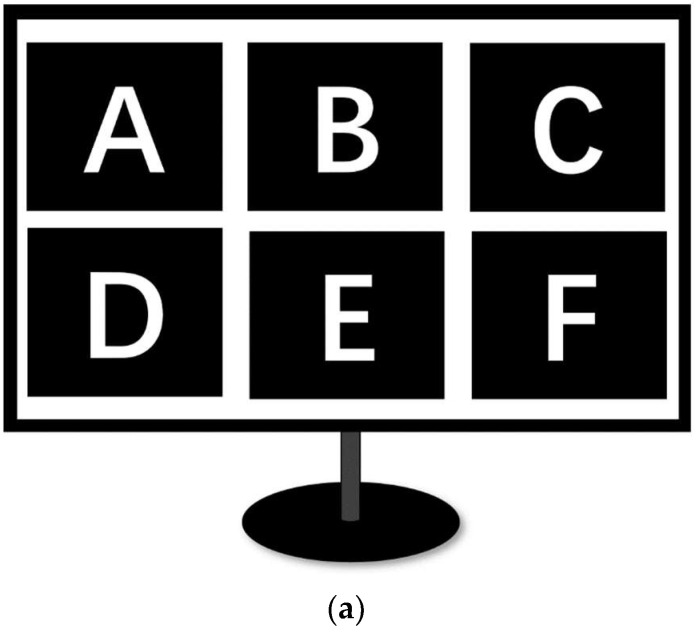

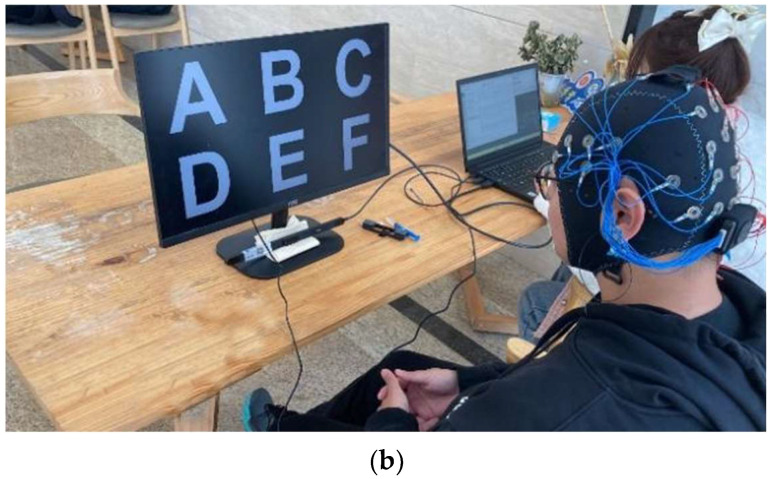
Stimulus interface (**a**) and experimental scene (**b**).

The design of the visual stimulator is an important experimental step to present flashing of lights. In this work, the programming platforms used for the creation of stimulus were MATLAB and Psychophysics Toolbox Version 3 (University of California, San Diego, United States), available at http://psychtoolbox.org/ (accessed on 17 October 2022). The user was instructed to follow the task paradigm presented on a 24-inch LCD monitor screen with a refresh rate of 60 Hz. A 60 Hz refresh rate implies that the time duration of each frame is 1/60 s. The color of frames reverses from black to white or white to black per cycle. Hence, the stimulation frequency can be determined by controlling the number of frames per cycle as f = 60/frame size. For instance, a 10 Hz stimulus is prepared when the frame color is black for three frames and white for the next three frames during one complete cycle; thus, there are a total of six frames per cycle. Adopting the same method, six stimuli of frequencies (8 Hz, 10 Hz, 12 Hz, 13 Hz, 14 Hz, and 15 Hz) were obtained. To avoid the coincidence of harmonics, the chosen flicker frequencies were not multiples of each other. The participants were sitting in an armchair 60 cm from the monitor. Figure 6 shows the experiment environment.

### 3.2. EEG Data

The dataset is freely available at Wesley1King/SSVEP-data: SSVEP data (github.com, accessed on 18 March 2023). It contains nine MATLAB MAT files corresponding to data for all subjects (approximately 44.8 MB in total). The data are stored in MATLAB matrices as double-precision floating-point values. These files are named subject indexes (i.e., S01.mat, …, S9.mat). For each file, loading the data in MATLAB generates a 4D matrix called “data” with dimensions (32, 1024, 6, 12). The four dimensions are Electrode Index, Time Point, Target Index, and Experiment Index. For a single trial, the data matrix consisted of 72 trials (6 targets × 12 blocks), each consisting of 1024 points of data in 32 channels. As described in the data preprocessing subsection, the data length was eight s (i.e., 8 × 128 = 1024 time points), basic bandpass filtering from 1–60 Hz, and ICA treatment of EEG to remove ocular and EMG artifacts. The “Readme.txt” file explains the data structure and other task-related information.

## 4. Results

### 4.1. Test Results Based on EEG from Public Dataset

The RLS-CCA method was first tested on the public dataset of Tsinghua University. Experiments showed that the RLS adaptive filtering CCA method proposed in this paper is significantly superior to the traditional CCA method.

In addition to the recognition accuracy, the performance of the SSVEP brain–computer interface could also be evaluated by the information transfer rate (ITR), which (in bits/min) can be estimated as follows [42]:

(10) ITR=log2M+Plog2P+1−Plog21−PM−1×60T

where M is the number of classes, P is the accuracy of target identification, and T (seconds/selection) is the average time for a selection.

The source of SSVEP signals were mainly recorded from the occipital region (Pz, PO5, PO3, POz, PO4, PO6, O1, Oz, O2). We performed detailed comparative experiments, testing, respectively, the selection of different electrode channel combination, the basic CCA algorithm, and the RLS-CCA method accuracy rate, and information transmission rate details are shown in Table 1 and Table 2. Table 1 shows the experimental data for comparing the number of different nonoccipital electrode channels when determining the occipital electrode. Table 2 shows the experimental results of determining the number of nonoccipital electrode channels and adding a group of occipital electrode channels to reduce the number of occipital electrode channels to three and the number of nonoccipital electrode channels to five. Since we used publicly available datasets, here, we worked with the Standard-CCA and FBCCA methods [27] proposed by Chen et al. in 2015, which were compared in terms of accuracy and ITR. As their methods only have relevant research on 64 electrode channels, we compared the accuracy and ITR under 64 electrode channels, and the comparative data are shown in Table 3.

The results show that the accuracy rate and information transmission rate of the RLS-CCA are much higher than those obtained by the ordinary CCA algorithms, and there is almost no effect on the accuracy rate when the number of nonoccipital electrode channels is reduced.

**Figure 7:**
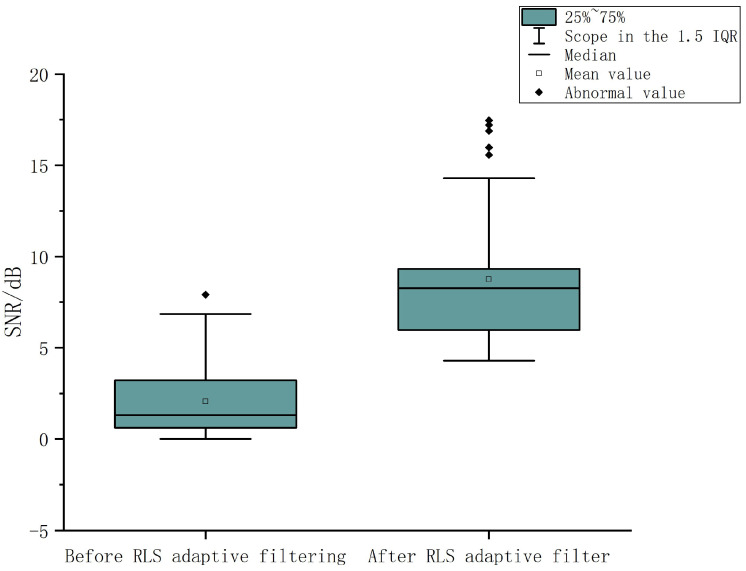
SNR of before and after RLS adaptive filtering.

The RLS adaptive filtering algorithm used in this paper is different from some empirical algorithms in theory. For example, wavelet transform requires a lot of training, which is an empirical algorithm that needs to find the mother wavelet function, but no general mother wavelet function can be applied to all signals [43]. The EMD method uses a shift process to decompose signals into time series, which is a fixed empirical pattern [44]. Many interference sources will generate interference noise, and RLS adaptive filtering estimates the interference from other noise sources. The distribution of signal-to-noise ratio (SNR) before and after RLS adaptive filtering is shown in Figure 7, showing a significant improvement in signal-to-noise ratio. For a specific frequency in an EEG signal, the expected SSVEP features are not filtered out, which is more accurate and has better robustness.

**Figure 8:**
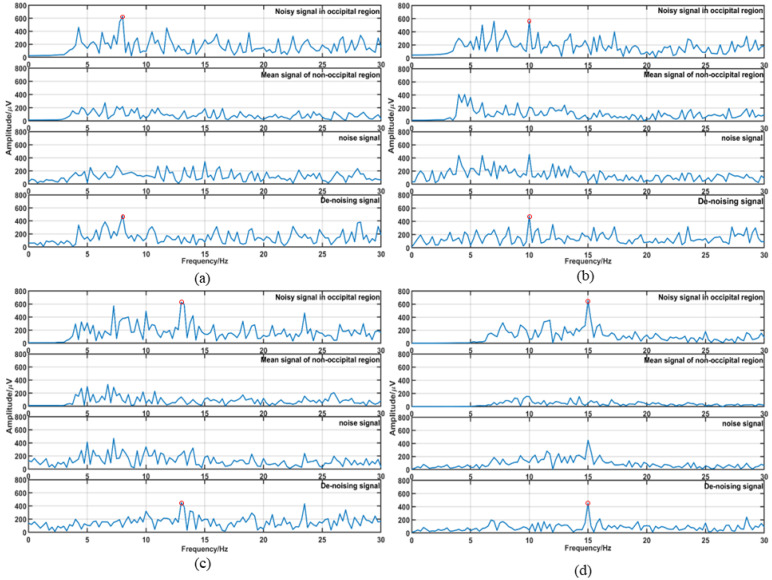

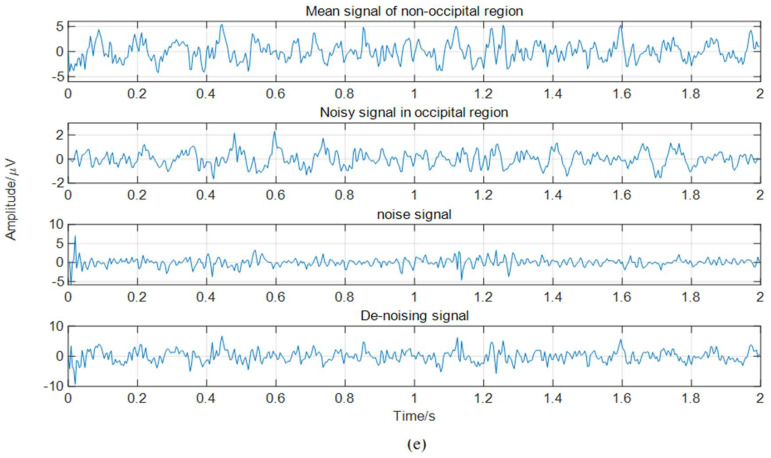
Filter result graph: (**a**–**d**) are the frequency-domain filtered images of annotated targets 8 Hz, 10 Hz, 13 Hz, and 15 Hz, respectively, and (**e**) is the time domain image of 8 Hz.

Figure 8 shows the RLS filtered frequency domain waveform of four subjects and the time domain waveform of subject no. 1. The following four images show all waveforms for subject gazing at 8 Hz (**a**), 10 Hz (**b**), 13 Hz (**c**), and 15 Hz (**d**) targets. The red circles marked the characteristic frequency of SSVEP. Figure 8e shows the time domain plot corresponding to Figure 8a. The four columns from top to bottom correspond to the input occipital signal, nonoccipital signal, noise signal, and denoised signal. In the frequency domain image, the noninduced SSVEP signal filtering is significant, and the relative amplitude of the induced SSVEP component increases.

### 4.2. Test Results Based on Actual Experiment

The data collected by the experiment were basically processed by EEGLAB, and with the Tobii Eye Tracker, it was possible to detect visual evoked potential events asynchronously. The classification of experimental data also used a 12-fold cross-witness method to obtain more reliable data results, and detailed results are provided in Table 4 and Table 5.

**Figure 9:**
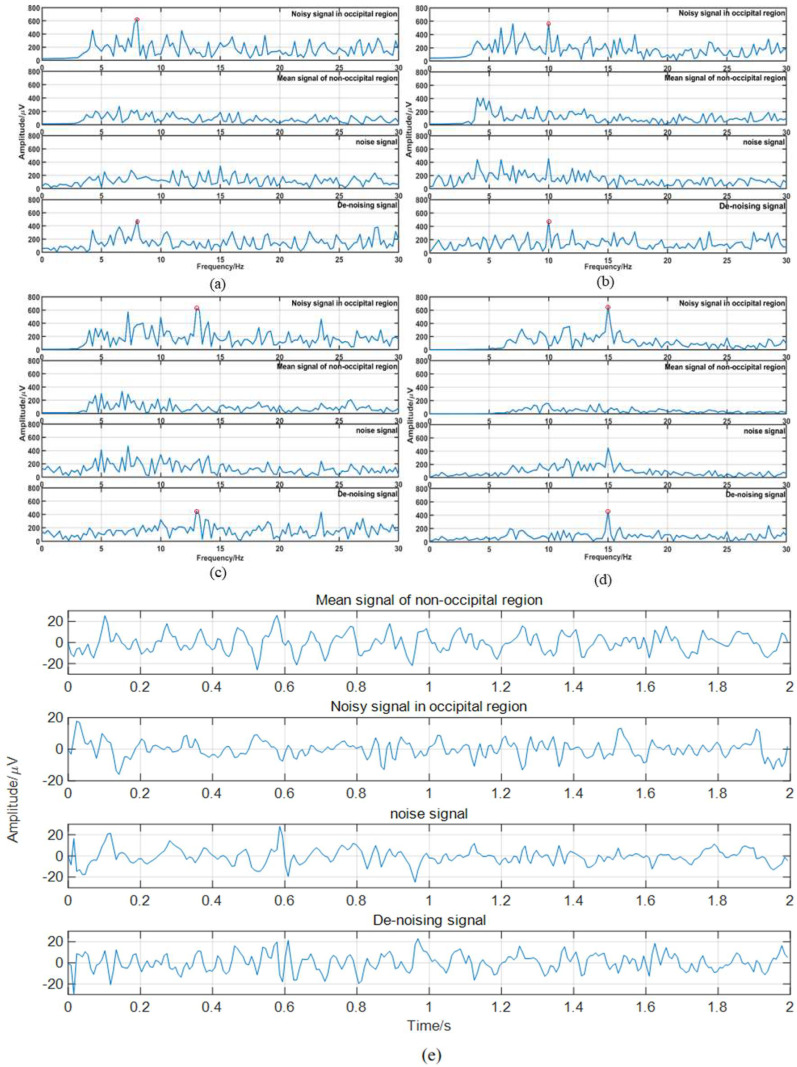
Filter result graph: (**a**–**d**) are the frequency-domain filtered images of annotated targets 8 Hz, 10 Hz, 13 Hz, and 15 Hz, respectively, and (**e**) is the time domain image of 8 Hz.

The results of the actual experiment show that the information transmission rate was reduced due to the reduction in the number of targets, but the accuracy rate was further improved compared to that of the synchronous experiment of the public dataset. A total of 100% accuracy was achieved in the experiment when all 32 channels were used. When using eight electrode channels (three pillow electrode channels and five nonpillow electrode channels), the accuracy reached 91.23. Figure 8 and Figure 9 are arranged in the same way. They show that the SSVEP characteristics are more significant after the RLS filter.

## 5. Conclusions

Traditional algorithms for processing EEG signals often differentiate signal components based on their frequency range. However, in reality, different EEG components from different sources often have a lot of overlapping frequency ranges, which can reduce the significance of SSVEP signals. In this paper, based on the hypothesis that EEG signals are the mixture of electrical signals from different nerve centers under the action of volume transmission, we used RLS principles to estimate the common components of EEG signals from occipital and other parts. These common parts can be considered as crosstalk from the nonevoked region to EEG signals generated by the visual central. By using an adaptive filter, we can remove this part of EEGs from the occipital EEG signals. As a result, when spontaneous EEGs or other interference are similar to the frequency of SSVEP signals, traditional frequency-based recognition methods can easily be interfered with, while this feature extraction algorithm based on RLS adaptive filtering can effectively eliminate them.

Compared with the standard CCA of FBCCA method, the accuracy of the RLS-CCA method was significantly improved on a public dataset. At the same time, the effectiveness of the method was also verified through actual experimental analysis. The accuracy rate reduced inconspicuously when the reference channels in the nonevoked region or the SSVEP recording channels in the occipital region were reduced. It shows that the RLS-CCA method causes EEG signals to be a stable information source. Based on this new SSVEP detection method, the features of SSVEP can also be extracted well in a short time window by using fewer electrodes; the SSVEP experiments could have fewer electrode channels and are more suitable for wearable environments.

## Figures and Tables

**Table 1:** Public dataset test results (9 OC channels).

Algorithm	ITR and Accuracy (ACC)	EEG Channel		
		9 OC Channels		
		5 Non-OC	30 Non-OC	55 Non-OC
CCA	ACC	66.88%	68.41%	69.91%
	ITR	83.19	84.23	84.87
RLS-CCA	ACC	93.94%	94.37%	94.41%
	ITR	139.85	142.22	143.35

**Table 2:** Public dataset test results (55 and 5 non-OC channels).

Algorithm	ITR and ACC	55 Non-OC Channels		5 Non-OC
		3 OC	6 OC	3 OC
CCA	ACC	55.33%	71.34%	56.97%
	ITR	59.40	87.31	60.53
RLS-CCA	ACC	81.67%	94.33%	82.14%
	ITR	108.87	141.96	110.67

**Table 3:** Comparison table of early algorithms.

Algorithm	ITR	ACC
Standard-CCA	105.50	65.20%
FBCCA	140.50	80.50%
RLS-CCA	143.35	94.41%

**Table 4:** Actual experimental test results (9 OC channels).

Algorithm	ITR and Accuracy (ACC)	EEG Channel		
		9 OC Channels		
		5 Non-OC	15 Non-OC	23 Non-OC
CCA	ACC	20.57%	23.82%	26.75
	ITR	0.33	0.43	1.01
RLS-CCA	ACC	96.59%	97.34%	100%
	ITR	57.57	60.14	61.98

**Table 5:** Actual experimental test results (23 and 5 non-OC channels).

Algorithm	ITR and Accuracy (ACC)	23 Non-OC Channels		5 Non-OC
		3 OC	6 OC	3 OC
CCA	ACC	11.87%	19.45%	12.54%
	ITR	0	0.23	0
RLS-CCA	ACC	91.20%	100%	91.23%
	ITR	47.17	61.95	48.3

## Data Availability

The dataset is freely available at Wesley1King/SSVEP-data: SSVEP data (github.com, accessed on 18 March 2023).

## Abbreviations

CCA	Canonical correlation analysis
SSVEPs	Steady-state visual evoked potentials
BCI	Brain–computer interface
SNR	Signal-to-noise ratio
EEG	Electroencephalographic
RLS	Recursive least squares
RLS-CCA	The CCA-based integrated RLS filter algorithm
CNS	Central nervous system
PSDA	Power spectral density analysis
FBCCA	Filter bank canonical correlation analysis
CNN	Convolutional neural network
AR-SSVEP	Augmented reality steady-state visual evoked potential
CAR	Common average reference
THU	Tsinghua University
JFPM	Joint frequency and phase modulation
ICA	Independent component analysis
LCD	Liquid crystal display
ACC	Accuracy
ITR	Information transfer rate
OC	Occipital electrode channels
non-OC	Nonoccipital electrode channels
